# PANoptosis-related genes function as efficient prognostic biomarkers in colon adenocarcinoma

**DOI:** 10.3389/fendo.2024.1344058

**Published:** 2024-03-04

**Authors:** Yang Liu, Yizhao Wang, Huijin Feng, Lianjun Ma, Yanqing Liu

**Affiliations:** ^1^ Endoscopy Center, China-Japan Union Hospital of Jilin University, Changchun, Jilin, China; ^2^ Herbert Irving Comprehensive Cancer Center, Columbia University, New York, NY, United States

**Keywords:** PANoptosis, prognostic model, colon adenocarcinoma, tumor immunity, tumor microenvironment, risk score

## Abstract

**Background:**

PANoptosis is a newly discovered cell death type, and tightly associated with immune system activities. To date, the mechanism, regulation and application of PANoptosis in tumor is largely unknown. Our aim is to explore the prognostic value of PANoptosis-related genes in colon adenocarcinoma (COAD).

**Methods:**

Analyzing data from The Cancer Genome Atlas-COAD (TCGA-COAD) involving 458 COAD cases, we concentrated on five PANoptosis pathways from the Molecular Signatures Database (MSigDB) and a comprehensive set of immune-related genes. Our approach involved identifying distinct genetic COAD subtype clusters and developing a prognostic model based on these parameters.

**Results:**

The research successfully identified two genetic subtype clusters in COAD, marked by distinct profiles in PANoptosis pathways and immune-related gene expression. A prognostic model, incorporating these findings, demonstrated significant predictive power for survival outcomes, underscoring the interplay between PANoptosis and immune responses in COAD.

**Conclusion:**

This study enhances our understanding of COAD’s genetic framework, emphasizing the synergy between cell death pathways and the immune system. The development of a prognostic model based on these insights offers a promising tool for personalized treatment strategies. Future research should focus on validating and refining this model in clinical settings to optimize therapeutic interventions in COAD.

## Introduction

Maintenance of homeostasis is crucial for mammals, as multicellular organisms, whether they are in favorable environments or facing harsh conditions (1). A key method to achieve this involves balancing cell proliferation and death. Cell death, when occurring in an appropriate spatio-temporal manner, is not necessarily detrimental to the host. The discovery of apoptosis decades ago (2) revealed that certain forms of cell death are not merely passive processes but are intricately regulated, introducing the concept of ‘regulated cell death’ (RCD). Following apoptosis, additional RCD types such as necroptosis, pyroptosis, ferroptosis, and autophagy-dependent cell death have been identified, with over ten distinct types recognized to date (3). These cell death types function together to orchestrate normal development, respond to extracellular or intracellular stresses, and also have important roles in diverse diseases. Among them, apoptosis, necroptosis, and pyroptosis are the most extensively studied, with their molecular mechanisms and regulations well-characterized. Furthermore, the critical physiopathological relevance of these three cell death modes has been investigated in detail.

Apoptosis is the first discovered RCD, and its name was coined more than 50 years ago (4). Now we know that apoptosis is composed by intrinsic and extrinsic pathways (5). Intrinsic apoptosis is initiated by the leakage of mitochondrial apoptogenic substances (like cytochrome c) to the cytoplasm. This process is regulated by the B-cell lymphoma-2 (BCL-2) protein family members. Cytochrome c then activates a caspase protein family member caspase 9 (CASP9). Activated CASP9 can activate the downstream CASP3/6/7, which function as apoptosis executioners to kill the cell. Extrinsic apoptosis is triggered by the binding of ligand to the death receptors [Fas cell surface death receptor (FAS), and TNF receptor superfamily members (TNFRSFs)]. Then adapter proteins Fas-associated death domain (FADD) and TNF receptor-associated death domain (TRADD) are recruited to the death receptors to activate CASP8 and CASP10, which can then activate CASP3/6/7 to cause cell death (5). It was long believed that neither pathways of apoptosis lead to immune response. However, discoveries in recent years argue against this viewpoint (6). The observation of pyroptotic cell death was recorded as early as 1980s (7, 8). It was until 2001, Brad Cookson and Molly Brennan proposed this term pyroptosis (9). Pyroptosis is now recognized a significant RCD involved in innate immunity. It is mainly triggered by pathogen-associated molecular patterns (PAMPs) and danger-associated molecular patterns (DAMPs), like LPS. In human, PAMPs and DAMPs activate some caspase proteins CASP1/4/5 (in mouse, they are CASP1 and CASP11). Caspase and other proteins assembly into inflammasome complex to activate gasdermin D/E (GSDMD/E) to form pore on cell membrane and make the cell die. Meanwhile, the cell will secrete IL-1β and IL-18 to promote inflammation, helping the host to defend against the pathogen infection (10). Necroptosis was named by Yuan et al. in 2005 to describe a cell death resembling necrosis but is regulated or programed (11). Unlike apoptosis and pyroptosis, necroptosis is independent of caspase proteins. It is mainly mediated by receptor-interacting serine/threonine-protein kinase 1 (RIPK1), RIPK3, and mixed lineage kinase domain-like protein (MLKL). Upon stresses like virus infection, toxin, and injury, RIPK1 is activated to bind RIPK3 to form a complex—necrosome, which can phosphorylate and activate MLKL, resulting in cell death (12). In summary, each of these three RCDs has its own triggers, molecular mechanisms, and physiopathologic importance. Researchers once thought that these cell death modes function separately. One cell, at a specific condition, should adopt only one cell death mode to die. However, the discovery of PANoptosis changed this idea.

In 2016, when studying the host immune response to influenza A virus (IAV) infection, Thirumala-Devi Kanneganti lab observed that IAV proteins NP and PB1 can be sensed by Z-DNA binding protein 1 (ZBP1). Then ZBP1 activates NLR family pyrin domain containing 3 (NLRP3)-dependent inflammasome via RIPK1–RIPK3–CASP8 axis. This signaling pathway eventually results in cell death. Interestingly, this cell death exhibits mixed features of apoptosis, necroptosis, and pyroptosis (13). To describe this novel cell death modality that cell simultaneously undergoes Pyroptosis, Apoptosis, and Necroptosis, Thirumala-Devi Kanneganti and colleagues proposed a new term PANoptosis in 2019 (14). Since then, many labs independently demonstrated that PANoptosis is a new type of inflammatory cell death mode. It is part of the innate immune response to diverse pathogen (including bacteria, fungus, and virus) infections. The key to PANoptosis is the assembly of PANoptosome. There are different types of PANoptosomes. Briefly, it is made up of PAMP or DAMP sensors (including ZBP1, AIM2, and NLRP3), adaptors (like ASC and FADD), and enzymatic effecters (such as RIPK1, RIPK3, and CASP8). The PANoptosome would transduce the pathogenic signal to the terminal cell death executioners to carry out pyroptosis, apoptosis, and necroptosis. Along with the demise of cell, many inflammatory cytokine will be released to promote inflammation (15, 16). To date, PANoptosis has been found to be related to lots of disorders, including pathogen infection (17–19), tissue injury (20, 21), and even cancer (22, 23). The association between PANoptosis and cancer is not surprising, as apoptosis, pyroptosis, and necroptosis are all highly related to cancer. Moreover, targeting these cell death pathways have shown promising effects in cancer treatment (24–26). Although there are studies demonstrating that identifying PANoptosis patterns in cancer can predict survival and response to immunotherapy and chemotherapy, and regulating PANoptosis-related genes and proteins can promote cancer cell death and improve therapy outcomes, now the research on PANoptosis and cancer is just in its infancy (27). Given this, it is necessary to investigate the potential association between PANoptosis and cancers.

In this current study, we use colon adenocarcinoma (COAD) as a model to explore the role of PANoptosis-related genes in cancer. We successfully identified two genetic subtype clusters in COAD, marked by distinct profiles in PANoptosis pathways and immune-related gene expression. A prognostic model based on PANoptosis-related genes, incorporating these findings, demonstrated significant predictive power for survival outcomes, underscoring the interplay between PANoptosis and immune responses in COAD.

## Methods

### Data collection and processing

We initiated our research by acquiring a comprehensive dataset of transcriptome sequencing and clinical data from The Cancer Genome Atlas-Colorectal Cancer (TCGA-COAD) repository. The dataset included 458 unique patient cases, comprising 41 normal tissue samples and 483 COAD tissue samples. Strict quality control was implemented to ensure the reliability and consistency of the procured samples, excluding the datasets with incomplete records or ambiguous clinical outcomes. For a thorough investigation of PANoptosis, we incorporated five distinct pathways from the Molecular Signatures Database (MSigDB) version 7.4 (GSEA | MSigDB: gsea-msigdb.org). These pathways included REACTOME_PYROPTOSIS, HALLMARK_APOPTOSIS, KEGG_APOPTOSIS REACTOME_APOPTOSIS, and KEGG_NECROPTOSIS (**Supplementary Table S1**). Additionally, based on earlier studies, we identified a set of immune-related genes that are pivotal to our analysis (28).

### Single-sample gene set enrichment analysis of PANoptosis-related and immune-related genes

The interaction between PANoptosis and the immune system in cancer has been previously reported (29, 30). To obtain a clearer understanding of this interaction, the transcriptome data were analyzed using single-sample gene set enrichment analysis (ssGSEA) to generate enrichment scores for PANoptosis-related and immune-related genes. This process was conducted using the GSVA package in R (31). ssGSEA is a widely used method to assess gene set enrichment within individual samples, which allows the identification of specific molecular signatures.

### Correlation analysis and clustering

To investigate the potential association between PANoptosis and immune response, we performed a correlation analysis between enrichment scores of PANoptosis-related and immune-related genes using the ‘cor.test’ function in R. A strong correlation between immune features and PANoptosis might suggest that these processes are interconnected in initiation and progression of COAD. For the strongly correlated features (Spearman correlation coefficient greater than 0.7), we utilized hierarchical clustering using the ‘hclust’ function in R (32). This technique groups samples exhibiting similar patterns, enabling the assessment of distinct molecular subtypes. Strong correlations reveal co-expression between immune response and PANoptosis-related genes, while clustering identifies co-expressed gene groups.

### Analysis of differential gene expression

Characterizing the differences in gene expression between identified clusters can provide vital insights into the underlying biological pathways. Differential gene expression analysis between the clusters was performed using the ‘limma’ package in R (33), with a cutoff of logFC = 0.5 and p-value < 0.05.

### Prognostic gene identification

The survival analysis for PANoptosis genes was performed using the ‘survival’ and ‘survminer’ packages in R. By identifying genes associated with survival differences, we can gain valuable information on potential prognostic biomarkers. The Cox proportional hazards model was applied for univariate survival analyses. This analysis enable the investigation of the relationship between gene expression levels and survival outcomes, providing essential information for prognostic assessment.

### Intersection of differential and prognostic genes and consensus clustering

We aimed to identify the genes that are both differentially expressed in the immune-related groups and associated with survival. The intersection of differential genes and PANoptosis-related prognostic genes was calculated. To further explore the molecular subtypes for better clinical assessment, we used consensus clustering using the ‘ConsensusClusterPlus’ package in R with the intersected gene set. The maximum number of clusters (K) to test was set at 9. A total of 50 resampling iterations were performed for each K. In each iteration, 80% of the samples (pItem=0.8) were randomly selected.

### Analysis of tumor mutation burden and immune landscape

Tumor mutation burden (TMB) is a measure of the number of somatic mutations present in a tumor. Higher TMB levels correlate with a better immunotherapy response, making it a valuable metric for assessing treatment potential. The TMB for each sample group was calculated using the maftools package in R based on the mutation data. The ‘ESTIMATE’ package in R was used to infer the stromal and immune scores, reflecting the immune landscape. Understanding the immune landscape within tumors allows for improved tumor characterization and prediction of response to immunotherapies.

### Immune escape analysis

Tumor immune escape is a significant concern when considering immunotherapy treatment strategies. To predict the response to immune checkpoint blockades, we utilized the web tool Tumor Immune Dysfunction and Exclusion (TIDE, http://tide.dfci.harvard.edu/), which calculates immune evasion scores based on gene expression profiles.

### Single-cell transcriptomic analysis

To explore the cellular context of genes constituting the identified biomarker signature, single-cell transcriptomic data was analyzed using The Tumor Immune Single-Cell Hub (TISCH, http://tisch.comp-genomics.org). By investigating the gene signature expression in different cell types, we can extract valuable biological insights into how these genes may functionally affect the tumor microenvironment.

### Establishment of prognostic model using LASSO cox regression

Constructing a prognostic model for clinical applications is critical for patient stratification and treatment decision-making. The Least Absolute Shrinkage and Selection Operator (LASSO) method was utilized to minimize overfitting during the establishment of the prognostic model using the ‘glmnet’ package in R (34). Additionally, we employed Cox regression to evaluate relationships between survival time and predictors. This approach provides a robust and interpretable model, allowing clinicians to evaluate patients’ risk profiles.

### Receiver operating characteristic curve and calibration curve analysis

We assessed the predictive accuracy of our risk score model using the ROC curve (‘pROC’ package in R) and Calibration curve (‘rms’ package in R) analyses (35). These methods offer quantitative measurement of our proposed model’s performance and allow comparison with other clinical factors.

### Functional enrichment analysis

To enhance our understanding of the biological processes and pathways altered within the identified gene sets, differentially expressed genes were subjected to both GO (Gene Ontology) and KEGG (Kyoto Encyclopedia of Genes and Genomes) enrichment analyses using the clusterProfiler package in R (36). These approaches provide a systematic representation of the biological functions modulated by the genes of interest.

### Nomogram construction and calibration curve validation

A nomogram was constructed, integrating the risk score and other relevant clinicopathological factors, to provide a comprehensive representation for the prediction of individualized survival probability. The nomogram was built using the ‘rms’ package in R. Moreover, the performance of the nomogram was further assessed by plotting calibration curves. Calibration curves offer a visual comparison between the predicted and observed outcomes, providing a direct evaluation of the accuracy of the nomogram.

## Results

### Single-sample gene set enrichment analysis of PANoptosis-related and immune-related genes

**Figure 1:**
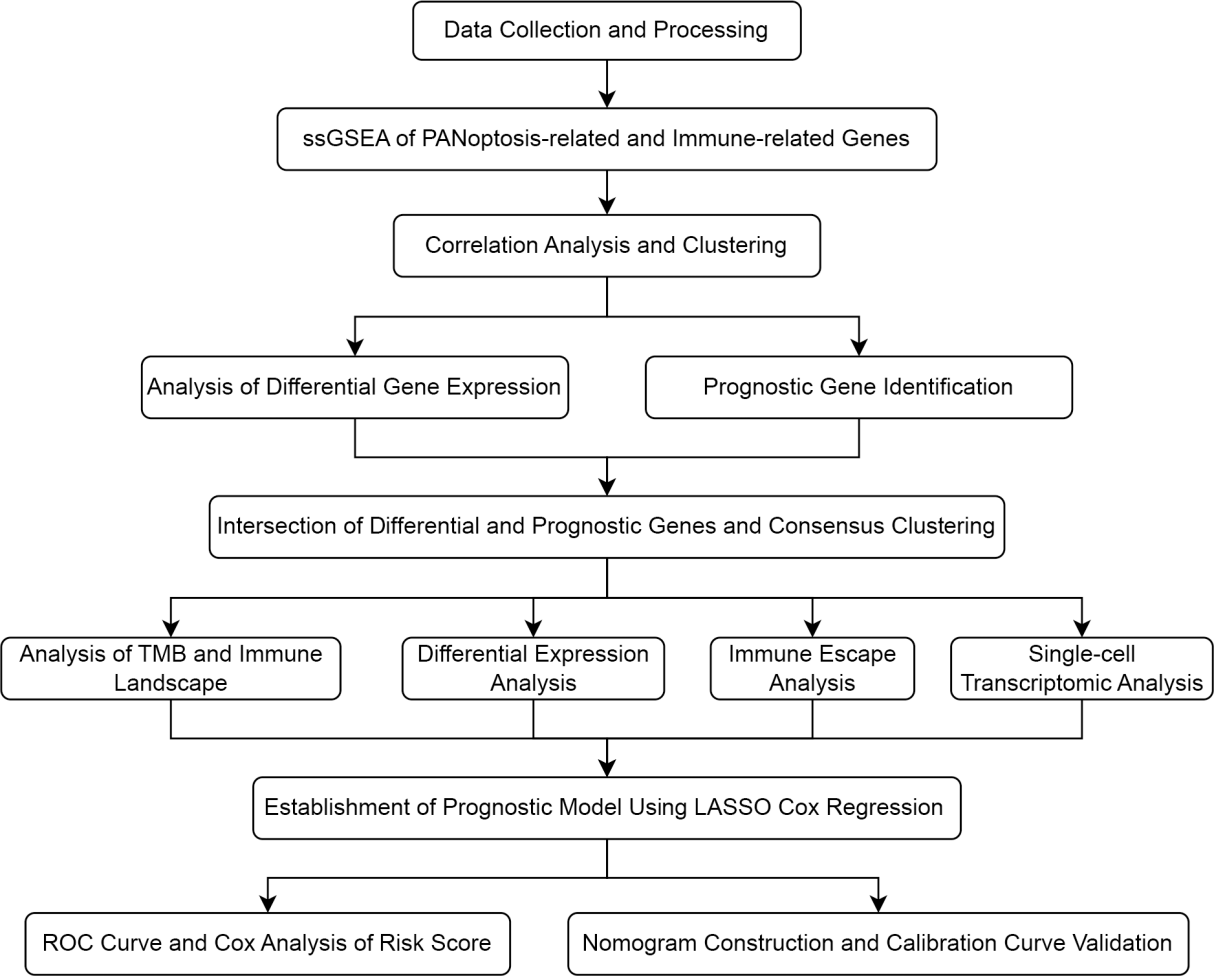
A flow chart of the entire study.

**Figure 2:**
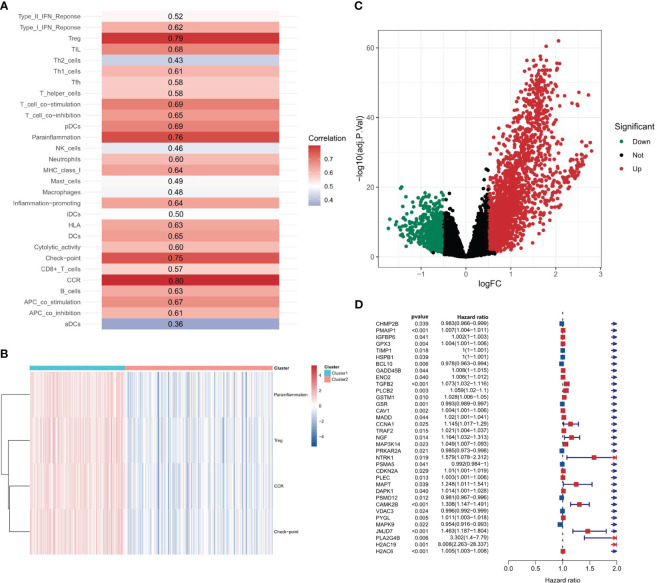
Correlation between PANoptosis and immune. **(A)** Spearman correlation coefficients depicting associations between the enrichment scores of PANoptosis-related and immune-related genes. **(B)** The distribution of immune feature gene set enrichment scores across two stratified clusters. **(C)** Volcano plot displaying differential gene expression between two clusters.

The study flowchart is depicted in **Figure 1**. In this study, we first performed a comprehensive Single-Sample Gene Set Enrichment Analysis (ssGSEA) for the PANoptosis gene set and immune-related gene set, providing an integrated view of the PANoptosis and immune landscape in the COAD sample set. We observed a strong correlation (Spearman correlation coefficient > 0.7) between the enrichment scores of the PANoptosis gene set and four immune feature gene sets (Treg, parainflammation, CCR, and immune checkpoint), as depicted in **Figure 2A**. The strong correlation indicates a likely co-regulation or mutual influence. Immune responses are intricately tied to PANoptosis, a process that maintains cellular homeostasis. For instance, Tregs, known to suppress immune responses, might be leveraging PANoptosis to maintain immune tolerance (37). Parainflammation, a response to tissue stress, could be interlinked with PANoptosis as a means of removing damaged cells and promoting tissue repair (38). CCR and immune checkpoint genes, key mediators of immune responses, might be influencing or influenced by apoptotic processes, contributing to the immune landscape (39, 40). However, the precise mechanisms remain to be elucidated and warrant further investigation.

### Hierarchical clustering based on strongly correlated immune-related gene set enrichment scores

Upon identifying immune feature gene sets with a Spearman correlation coefficient greater than 0.7, hierarchical clustering was performed. This resulted in the categorization of 189 samples into cluster 1 and 294 samples into cluster 2. The distribution of the enrichment scores for the four immune-related gene sets - Treg, Parainflammation, CCR, and checkpoint - across the two clusters is depicted in **Figure 2B**. Notably, all these scores were significantly higher in cluster 2, implying a heightened immune response or activity in this cluster. The clear separation into two clusters suggests inherent molecular differences between them.

### Intersection of differential and prognostic genes

**Figure 3:**
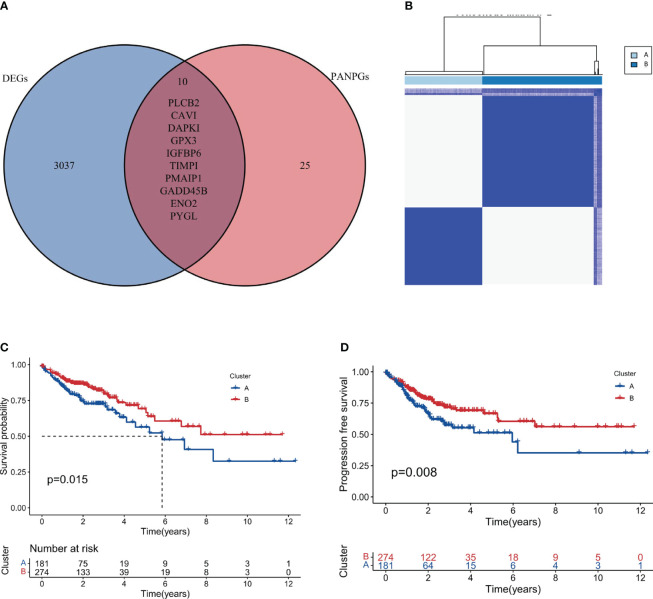
Intersection and clustering of PANoptosis-related prognostic genes. **(A)** Venn diagram showing the intersection of differentially expressed genes and PANoptosis-related prognostic genes. **(B)** Consensus clustering when k=2 indicating the optimal partition of samples into two distinct clusters, Cluster A and Cluster B. **(C, D)** Overall and progression-free survival analysis for the two clusters.

A differential gene expression analysis was performed between the two clusters with selection criteria of logFC= 0.5 and p-value< 0.05. As visualized in the volcano plot (**Figure 2C**), 2307 genes exhibited higher expression in cluster 1 (characterized by high immune feature gene set enrichment scores), while 741 genes were upregulated in cluster 2. The greater number of differentially highly expressed genes in cluster 1 can be attributed to the pronounced immune activity and potential interplay with various cellular pathways. A survival analysis of the PANoptosis gene set was executed using single-gene Cox analysis. The forest plot, depicted in **Figure 2D**, highlights the significance of certain PANoptosis genes in relation to patient survival. The intersection between the differential gene set and the survival-related PANoptosis genes yielded 10 genes of interest: PLCB2, CAV1, DAPK1, GPX3, IGFBP6, TIMP1, PMAIP1, GADD45B, ENO2, and PYGL. In light of previous scientific inquiries, it’s compelling to mention that many of the genes within this list of 10, specifically CAV1, DAPK1, GPX3, IGFBP6, TIMP1, GADD45B and ENO2 have been found associated intimately with colorectal cancer, as documented by several research studies (41–47). These genes, thereby, are candidates for further investigation concerning their role in COAD progression and therapeutic potential. This intersection is graphically represented in **Figure 3A**.

### Consensus clustering based on intersection genes

Based on the intersection genes identified in the previous step, consensus clustering was conducted. **Figure 3B** showcases the clustering results, and it was observed that the optimal classification was achieved when k=2. Consequently, 181 samples were allocated to cluster A, while 277 samples were designated to cluster B. It is worth noting that these two distinct territories, or clusters, may represent two different biological subtypes within the vast field of colorectal cancer respectively. Each subtype, potentially, could have unique implications for disease progression and prognosis. This result could enhance our molecular knowledge of colorectal cancer, potentially leading to more precise disease classification and individualized treatment strategies.

### Survival analysis of consensus clusters

Survival analysis, a cornerstone in oncological research, was employed to fathom the prognostic implications of our identified clusters. The results, illustrated in **Figures 3C** (Overall Survival) and **3D** (Progression-free Survival), clearly indicate that cluster B may represent a biological subtype associated with lower disease progression risk and better survival outcomes. Such a discovery could provide important insights for clinical decision-making and patient management.

### Tumor mutation burden analysis of clusters A and B

**Figure 4:**
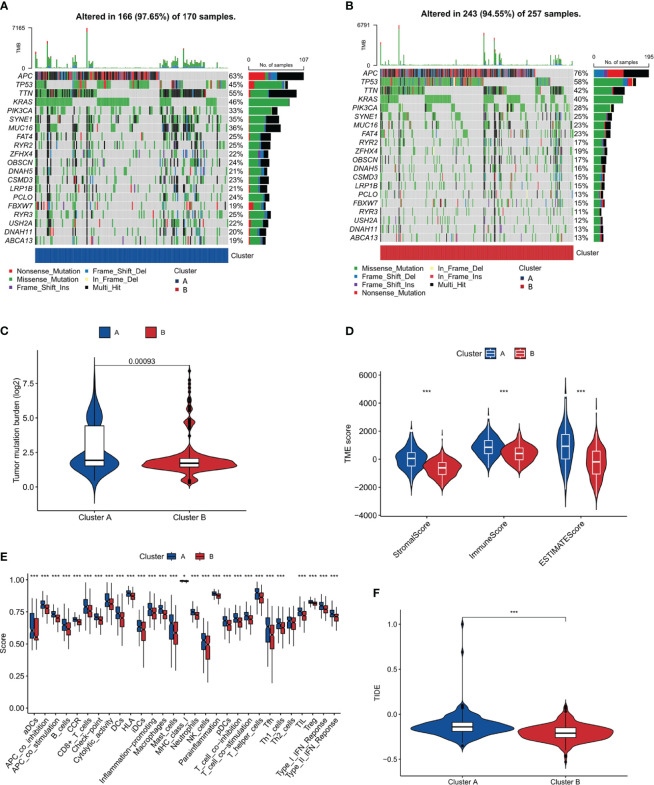
Tumor Mutation Burden and Immune Microenvironment Analysis. **(A, B)** Comparative plots of tumor mutation burden across the two clusters. **(C)** Plot showing higher tumor mutation burden in Cluster A compared to Cluster B. **(D)** Immune microenvironment scores (stromal, immune, and ESTIMATE scores) comparison between the clusters. **(E)** Analysis of immune cell infiltration showing a higher degree in Cluster A. **(F)** TIDE score analysis indicating a higher potential for immune escape in Cluster A. *, p<0.05; ***, p<0.001.

To delve deeper into the genetic landscape of the tumors, we embarked on an analysis of the tumor mutation burden (TMB) across the two clusters. TMB, a measure of the number of mutations within a tumor genome, has been spotlighted for its potential prognostic value, especially in the realm of immunotherapy. **Figures 4A**, **B** offer a comparative view of the TMB across clusters A and B. Intriguingly, cluster A, as accentuated in **Figure 4C**, boasted a higher TMB. Additionally, we observed that cluster B had a higher frequency of APC and TP53 mutations compared to cluster A. Conversely, cluster A had a higher frequency of TTN and RYR3 mutations compared to cluster B. These findings suggest that these two subtypes seem to have different genetic mutations, which could have unique implications for disease progression and prognosis. Based on the distinct mutation frequencies of genes, we can take specific treatments for different subtypes of COAD, improve diagnostics, hone immunotherapy precision and refine clinical trial designs.

### Immune microenvironment analysis of clusters A and B

An in-depth analysis of the immune microenvironment of the two clusters was conducted. **Figure 4D** reveals that cluster A had elevated stromalScore, immuneScore, and ESTIMATEScore compared to cluster B, indicating a more pronounced immune and stromal activity in cluster A. The ESTIMATEScore, a combination of the stromalScore and immuneScore, gives an overall impression of the tumor purity. The reason for conducting such an analysis is to gain insight into the potential mechanisms of immune response within these clusters. The elevated values in cluster A may suggest a more reactive immune environment, potentially leading to a more effective immune response against tumor cells. The results interestingly hint at cluster B having a better prognosis. This could be surprising at first glance, as a higher level of immune and stromal activity in cluster A could be interpreted as a more robust defense against cancer progression. However, this might not always be the case. The tumor microenvironment is a complex system where a high level of immune activity can sometimes be associated with immune evasion or suppression tactics used by cancer cells. Therefore, it seems that the less pronounced immune and stromal activity in cluster B might result in a better balance between tumor-suppressive and tumor-promoting activities, leading to a more favorable prognosis. This hypothesis aligns with the notion of ‘immunoediting’, a process that shapes the immunogenicity of the tumor (48). Further investigations are needed to validate these results and explore the underlying mechanisms that contribute to the apparent paradox. Additionally, in **Figure 4E**, our findings, which align with the observations above, show that in cluster A, the majority of these immune function scores were indeed upregulated. To further our understanding of the tumor’s interplay with the immune system, we turned our attention to the potential for immune escape. The Tumor Immune Dysfunction and Exclusion (TIDE) framework was employed for this purpose. Consistent with the anticipated outcomes from our preceding analysis, **Figure 4F**, showcasing our findings, revealed a heightened TIDE score for cluster A, implying a pronounced potential for immune escape.

### Single-cell transcriptomics analysis

**Figure 5:**
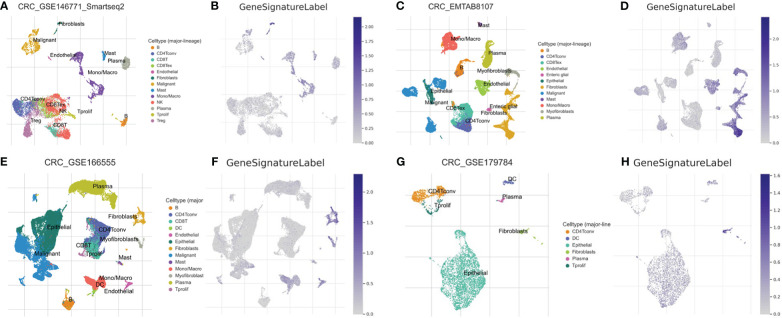
Single-Cell Transcriptomic Analysis. Expression analysis of a 10-gene signature in fibroblasts based on single-cell transcriptomics data in GSE146771 **(A, B)**, EMTAB8107 **(C, D)**, GSE166555 **(E, F)** and GSE179784 **(G, H)** database.

A single-cell transcriptomics analysis was conducted to delve deeper into the expression patterns of the 10-gene signatures. Interestingly, the 10-gene signature was observed to be highly expressed in fibroblast cells, as illustrated in **Figures 5A–H**. The high expression of these genes in fibroblast cells suggests that fibroblast cells may not only participate in the construction of the tumor microenvironment, but also play a role in regulating immune actions and cell death. It is intriguing, the recent studies indicate that fibroblasts hold a significant role in immune-related actions including immune escape and the process of PANoptosis, shedding light on the intricate connection of cellular mechanisms (49, 50). Additionally, the high expression of these genes may affect how these cells interact with tumor cells and other immune cells, further affecting the progression of colon cancer.

### Differential expression analysis between clusters A and B

**Figure 6:**
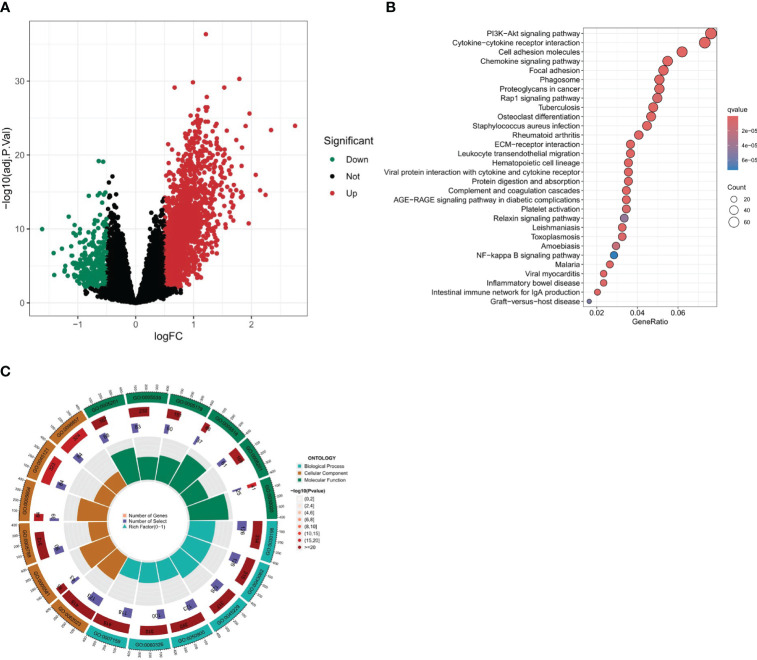
Differential expression analysis and enrichment analysis. **(A)** Volcano plot of differentially expressed genes between two clusters. **(B, C)** Enrichment analysis of differential gene set, including KEGG and GO enrichment analysis.

A differential analysis was undertaken between clusters A and B with a stringent selection criterion of logfc=0.5 and p-value<0.01. The volcano plot in **Figure 6A** reveals that 292 genes were downregulated in cluster A, while 1746 genes were upregulated.

### Enrichment analysis of differential gene set

To further our understanding of the biological underpinnings of our differential gene set, we embarked on a comprehensive enrichment analysis. This entailed both Gene Ontology (GO) and Kyoto Encyclopedia of Genes and Genomes (KEGG) enrichment analyses (**Figures 6B, C**). The intent was to categorize our genes into meaningful biological processes, molecular functions, cellular components, and pathways. According to the KEGG enrichment analysis, the following pathways were found to be significantly enriched: Cytokine-cytokine receptor interaction, Cell adhesion molecules, Chemokine signaling pathway, MAPK signaling pathway, Focal adhesion, and Calcium signaling pathway. These three pathways, namely, Cytokine-Cytokine Receptor Interaction, Chemokine Signaling Pathway, and Cell Adhesion Molecules (CAMs), are linked with immune activities, while the MAPK signaling pathway and the Cytokine-cytokine receptor interaction are closely related to PANoptosis. Additionally, it is interesting to note that many of these pathways are related to fibroblasts, which is consistent with our previous results. The Gene Ontology (GO) enrichment analysis revealed significant pathways across three main ontology categories. For Biological Processes, pathways such as extracellular matrix organization, extracellular structure organization, and leukocyte migration were predominant. In the Cellular Component category, collagen-containing extracellular matrix, collagen trimer, and membrane raft were highlighted. Meanwhile, the Molecular Function category emphasized pathways like extracellular matrix structural constituent and glycosaminoglycan binding. These findings provide key insights into the genetic basis of our study and set the stage for deeper investigations.

### Prognostic model construction based on lasso and cox regression

**Figure 7:**
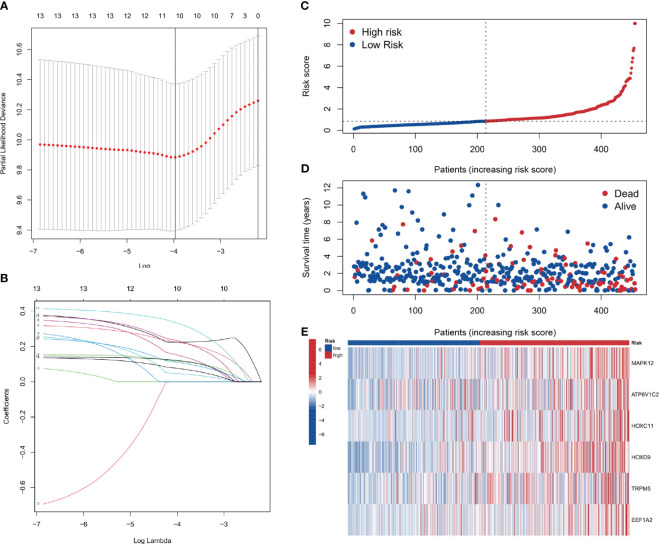
Prognostic model construction. **(A, B)** Plots for the selection of the optimal λ in the model. **(C, D)** Plots correlating patient risk scores with survival outcomes, indicating decreasing survival times with increasing risk scores. **(E)** The genes showing higher expression in the high-risk group.

Using the results from Lasso regression and Cox regression, a robust prognostic model was constructed. **Figures 7A**, **B**, representing the pathway diagram and deviation diagram respectively, aided in the optimal selection of the regularization strength λ (Lambda) value, which was determined to be 10. Finally, a risk score was constructed based on the expression levels of six genes: risk score=MAPK12 expression×0.375+ATP6V1C2 expression×0.231+HOXC11 expression×0.166+HOXD9 expression×0.224+TRPM5 expression×0.417+EEF1A2 expression×0.270.

### Patient risk stratification analysis

As depicted in **Figures 7C–E**, a correspondence is observed between the increment in risk scores and the reduction in patients’ survival time. Six genes, namely MAPK12, ATP6V1C2, HOXC11, HOXD9, TRPM5, and EEF1A2, show elevated expression in the high-risk group. The analysis shows that the risk score is a significant predictor of patient survival time. These genes may play a critical role in the development and progression of the disease and could be potential targets for therapeutic intervention.

### Risk score’s ROC curve analysis and cox analysis of risk score

**Figure 8:**
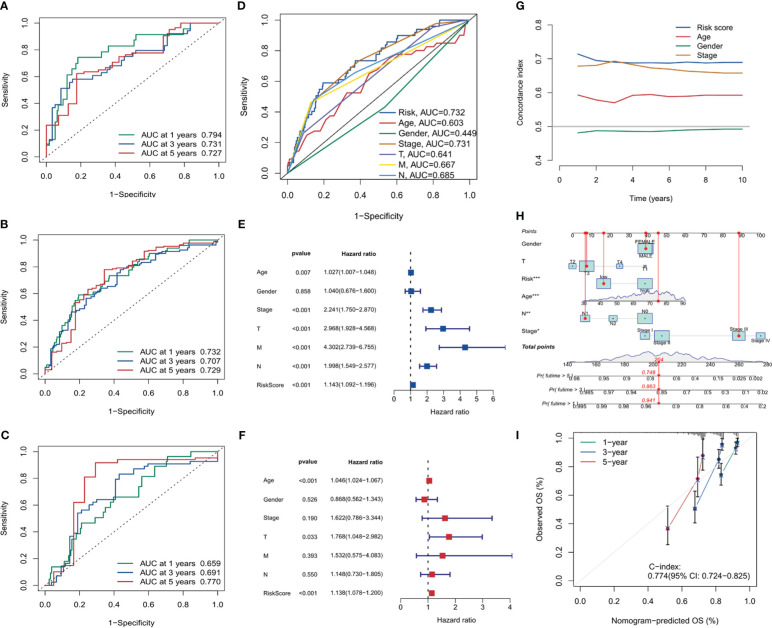
Risk score analysis and model validation. **(A–C)** Receiver operating characteristic (ROC) curves for risk scores in training, validation, and overall cohorts. **(D)** ROC curves comparing risk scores with clinical features. **(E, F)** Forest plots for univariate and multivariate Cox analysis showing the prognostic significance of risk scores. **(G)** The concordance curve and concordance index depicting a good agreement of the model. **(H)** Nomogram based on prognostic model and clinical traits. **(I)** Calibration curve for the nomogram. *, p<0.05; **, p<0.01; ***, p<0.001.

The ROC curve analysis for the risk score was conducted for the training set, validation set, and the entire sample set, as visualized in **Figures 8A–C**. In the entire group, 1-year AUC = 0.732, 3-year AUC = 0.707, and 5-year AUC = 0.729. In the present investigation, all AUC indicators surpass the benchmark of 0.7. This intimates a satisfactory discriminatory capacity of the risk score, providing a reasonably robust forecast of risk for future periods of 1 year, 3 years, and 5 years respectively. Additionally, **Figure 8D** presents the ROC curve correlating the risk score with clinical features, implying the risk score is a reliable predictor of future outcomes. Moreover, both univariate and multivariate Cox analyses were executed for the risk score. The forest plots in **Figures 8E**, **F** clearly demonstrate that the p-values for both analyses were less than 0.001, signifying the robustness of the risk score as a predictor.

To gauge the consistency and reliability of our risk score, we utilized the Consistency Index (C-index). **Figure 8G**, showcasing our findings, revealed a high C-index for our risk score, hinting at its reliability and accuracy.

In the final part of our analysis, we embarked on constructing a nomogram. This graphical tool offers clinicians a user-friendly interface to predict patient outcomes based on various factors. **Figures 8H**, **I** visually present the nomogram and its accompanying calibration curve. The calibration curve, a testament to the nomogram’s predictive accuracy with a C-index of 0.774, also further validates the efficacy of our prognostic model.

## Discussion

Colon cancer is now one of the major cancer types in digestive tract. COAD is the most popular colon cancer type, which causes thousands of death every year (51). Defeating this disease has long been a crucial goal for oncologists. To achieve this goal, now there are four main directions to pursue. The first one is to identify environmental and molecular risk factors for COAD and develop prevention methods for it. The second one is improving the screening and detection methods for COAD. The third one is deciphering the molecular mechanism for COAD initiation and development, based on which new therapeutics can be developed. The last one is to find out effective biomarkers for COAD prognosis, which can facilitate the choice of treatment regimen (52). Scientists and clinicians have made impressive progresses in all the four directions in the past decades, while there are also limitations and bottlenecks in each path. Further hope relies on the new progress in basic science and clinical practice about COAD. It is always important to refresh our understanding about COAD and effectively translate new knowledge into clinical application.

The discovery of PANoptosis sheds new light on COAD research. PANoptosis represents a quite novel cell death mode distinct from other cell deaths discovered before. Importantly, the three key components of PANoptosis (pyroptosis, apoptosis, and necroptosis) have all been demonstrated to be closely associated with COAD (53–55). Therefore, it is reasonable to speculate that the genes in the PANoptosis signaling pathway may have prognostic and therapeutic value in COAD. To explore this hypothesis, in this study we used clinical datasets to investigate the role of PANoptosis-related genes in COAD and made many interesting and important discoveries. Firstly, we revealed that PANoptosis gene signature is highly associated with immune cell activities, particularly the interferon pathway, Treg cells, NK cells, neutrophils, and macrophages. As PANoptosis is mainly induced in pathogen infection and the execution of PANoptosis releases many proinflammatory cytokines, the activation of host immune system is a consequent event following PANopotosis. It is worthy of noting that there are rich microbiota in the intestinal tract, which is relevant to COAD initiation (56). Intestinal tract is the frontier of defensing host from microbes infection. Importantly, the recent years identified intratumor microbiota in COAD (57). These facts raise the possibility that microbe infection may be a more frequent incident in COAD progression compared to other tumor types. That means PANopotosis and related immune response may be more easily triggered in COAD. What bacteria in the gut is more relevant to these events? How will this influence COAD progression and treatment? These are interesting directions to pursue. By overlapping the deregulated genes in COAD with PANoptosis-related genes, 10 genes (PLCB2, CAV1, DAPK1, GPX3, IGFBP6, TIMP1, PMAIP1, GADD45B, ENO2, and PYGL) stand out. The functions of PLCB2, ENO2, and PYGL in cell death have not been fully studied. CAV1 is suggested to regulate apoptosis and pyroptosis (58, 59). However, there is also papers showing that CAV1 participates in the modulation of ferroptosis (60, 61). Similar situation applies to GPX3, IGFBP6 and TIMP1 (62–64). These results raise a vital question that whether ferroptosis can be induced simultaneously with PANoptosis. More importantly, by using this ten gene signature, we can divide COAD patients into two different categories with distinct survival time, tumor mutation profile, tumor mutation burden, and tumor microenvironment. This gene set will benefit the classification, treatment option, and prognosis of COAD patients. Single-cell transcriptomics analysis showed that this ten-gene signature was highly expressed in fibroblast cells. Exploring deeper into the two categories of COAD patients, we identified many differentially expressed genes, with multiple functions. Then we constructed a more accurate and effective prognostic model based on these differential expressed genes. This model comprises 6 genes MAPK12, ATP6V1C2, HOXC11, HOXD9, TRPM5, and EEF1A2. While MAPK12, HOXC11, HOXD9, and EEF1A2 have been demonstrated to involve diverse tumors’ developments (65–68), their roles in COAD need to be elucidated in the future. The study on the function of ATP6V1C2 in cancer is rare. However, there is a paper revealing that ATP6V1C2 alone is a prognostic factor in COAD (69). Interestingly, TRPM5 has a role in taste transduction (70). It is also involved in metabolic disorders (71). How it is associated with COAD is not clear. Possible mechanism involves its cation channel feature, as tumor microenvironment is often acidic due to the active glycolysis (72). Overall, this 6-gene model is effective in COAD prognosis. One may argue that certain gene itself in this model has prognostic value in COAD (69). However, we think that this 6-gene combination may have a better and more accurate prognostic efficiency than a single gene in it. At present, there are not many researches using PANoptosis-related genes to predict the survival of tumor patients. This may be a promising direction to explore. Notably, this study, along with our several papers published before, reflects the usefulness of cancer database and multiomics in cancer research (73–75). These resources and methods, fueled by new basic findings (like PANoptosis), are now boosting the advance in oncology. However, future studies with an extensive clinical patient samples are required to further validate the current findings.

Several studies have explored the potential of targeting the PANoptosis pathway using specific medications for tumor treatment. A current example is Sulconazole, an FDA-approved antifungal drug. This medicine has been shown to induce PANoptosis in esophageal cancer cells by generating oxidative stress and hindering their ability to perform glycolysis (76). Its mechanism of action involves reducing hexokinase (HK) levels and inhibiting key signaling pathways such as PI3K/AKT, MEK/ERK, and STAT3. Furthermore, a recent study revealed that inhibiting the enzyme NFS1 or its phosphorylation can enhance the sensitivity of colorectal cancer cells to oxaliplatin by promoting PANoptosis, leading to improved chemotherapy outcomes (22).

Although PANoptosis shows great potential in COAD prognosis and treatment, the PANoptosis field faces lots of questions to be solved. Upon infection or stress, why does the cell induce three different cell death modes simultaneously? Maybe PANoptosis can ensue that infected or stressed cell can be eliminated efficiently. What is the exact mechanism underlying PANoptosis? How many pathogens or stimuli can activate PANoptosis? What is the molecular trigger of it? What is the detail for PANoptosome assembly? How many PANoptosomes exist? Now there is not a unified model for PANoptosis. What is the evolutional advantage of PANoptosis? What’s the advantage and disadvantage of PANoptosis compared with other cell death types? Is PANoptosis reversible, if the pathogen could be removed in time? Can PANoptosis spread to nearby cells? As PANoptotic cells can release different immunogenic cytokines, this activity may induce secondary PANoptosis in surrounding cells. Are there any other cell death type (for example ferroptosis) happening at the same time along with pyroptosis, apoptosis, and necroptosis when a typical PANoptosis-inducing pathogen invading the cell? What is the weight of relative contribution of the three branches (pyroptosis, apoptosis, and necroptosis) to PANoptotic cell death? The answer to this question should be pathogen, cell, and disease-specific. Sometimes, when one cell death type in PANoptosis is inhibited, other types of cell death may be enhanced (77). The three death modes work synergistically to kill the cell. How does PANoptosis cooperate with other cell death types to maintain the homeostasis of the host organism? Is there any effective marker for PANoptosis? This will benefit the molecular research on PANoptosis. A more important question is what is the pathological relevance of PANoptosis? How can we target PANoptosis to treat related disorders, particularly cancer? Inducing PANoptosis may have advantages over other cell death-inducing method to treat cancer, as PANoptosis is a mixed cell death type that may ensue the targeted cell will die more efficiently than triggering only one cell death type. Additionally, induction of PANoptosis will lead to immune cell activities, which may be leveraged to enhance the cancer treatment efficacy. How to specifically induce PANoptosis in tumor cell rather than normal cell? Is it possible to develop a PANoptosis-specific inhibitor or activator? A relevant question is that is there a master regulator of PANoptosis? p53 is such a candidate, as it mediates pyroptosis, apoptosis, and necroptosis, including other cell death modalities (78–81). When referring to PANoptosis in COAD, there are also many unaddressed issues. What triggers PANoptosis in COAD cell? When, where and how will PANoptosis happen in COAD patients? Is there a role for the intestinal microbiota on PANoptosis of COAD cell? Will PANoptosis influence the tumor microenviroment of COAD? As PANoptosis is highly pro-inflammatory, will PANoptosis affect the tumor immunity in COAD? Can we target PANoptosis to enhance tumor immunotherapy in COAD? How can we perform combined treatment of COAD by targeting PANoptosis and taking traditional therapies? Finding the answers to these questions requires involvement of more researchers and efforts in this field.

To sum up, we investigate the potential function of PANoptosis-related genes in COAD. We reveal that these genes are useful to classify COAD patients, which may help improve the treatment option for them. We also construct an effective prognostic model based on those genes. Our results indicate that PANoptosis may have crucial roles in COAD progression and therapy. We wish that more attentions and research/clinical efforts can be put into this field in the near future.

## Data availability statement

The original contributions presented in the study are included in the article/**Supplementary Material**. Further inquiries can be directed to the corresponding authors.

## Author contributions

YQL: Conceptualization, Writing – original draft. YL: Data curation, Formal analysis, Writing – original draft. YW: Data curation, Writing – review & editing. HF: Writing – original draft. LM: Conceptualization, Writing – review & editing.
